# Absorbable hydrogel spacer use in men undergoing prostate cancer radiotherapy: 12 month toxicity and proctoscopy results of a prospective multicenter phase II trial

**DOI:** 10.1186/1748-717X-9-96

**Published:** 2014-04-24

**Authors:** Matthias Uhl, Klaus Herfarth, Michael J Eble, Michael Pinkawa, Baukelien van Triest, Robin Kalisvaart, Damien C Weber, Raymond Miralbell, Danny Y Song, Theodore L DeWeese

**Affiliations:** 1 Department of Radiation Oncology, University of Heidelberg, Im Neuenheimer Feld 400, 69120 Heidelberg, Germany; 2 Department of Radiation Oncology, RWTH Aachen University, Aachen, Germany; 3 Department of Radiation Oncology, NKI-AVL Nederlands Kanker Instituut - Antoni van Leeuwenhoek, Amsterdam, The Netherlands; 4 Department of Radiation Oncology, Geneva University Hospital, Geneva, Switzerland; 5 Department of Radiation Oncology and Molecular Radiation Sciences, Johns Hopkins University School of Medicine, Baltimore, MD, USA

**Keywords:** Prostate cancer, Radiotherapy, Rectal toxicity, Hydrogel, Spacer, IMRT

## Abstract

**Background:**

Radiation therapy is one of the recommended treatment options for localized prostate cancer. In randomized trials, dose escalation was correlated with better biochemical control but also with higher rectal toxicity. A prospective multicenter phase II study was carried out to evaluate the safety, clinical and dosimetric effects of the hydrogel prostate-rectum spacer. Here we present the 12 months toxicity results of this trial.

**Methods:**

Fifty two patients with localized prostate cancer received a transperineal PEG hydrogel injection between the prostate and rectum, and then received IMRT to a dose of 78 Gy. Gastrointestinal and genitourinary toxicity were recorded during treatment and at 3, 6 and 12 months following irradiation by using the RTOG/EORTC criteria. Additionally, proctoscopy was performed 12 months after treatment and the results were scored using the Vienna Rectoscopy Scale (VRS).

**Results:**

Of the patients treated 39.6% and 12.5% experienced acute Grade 1 and Grade 2 GI toxicity, respectively. There was no Grade 3 or Grade 4 acute GI toxicity experienced in the study. Only 4.3% showed late Grade 1 GI toxicity, and there was no late Grade 2 or greater GI toxicity experienced in the study. A total of 41.7%, 35.4% and 2.1% of the men experienced acute Grade 1, Grade 2 and Grade 3 GU toxicity, respectively. There was no Grade 4 acute GU toxicity experienced in the study. Late Grade 1 and Grade 2 GU toxicity was experienced in 17.0% and 2.1% of the patients, respectively. There was no late Grade 3 or greater GU toxicity experienced in the study. Seventy one percent of the patients had a VRS score of 0, and one patient (2%) had Grade 3 teleangiectasia. There was no evidence of ulceration, stricture or necrosis at 12 months.

**Conclusion:**

The use of PEG spacer gel is a safe and effective method to spare the rectum from higher dose and toxicity.

## Introduction

Prostate cancer is the most common cancer in older men in the USA and Europe. The predicted 2013 prostate cancer death rate in Europe is 10.5/100.000 men. Despite treatment advances, prostate cancer still ranks third in cancer death, after lung and colon carcinoma [1]. Furthermore, the incidence of new prostate cancer cases in 2010 was the highest of cancers among men in developed countries across the globe [2]. Radiation therapy or prostatectomy are often the recommended treatment options for localized prostate cancer [3,4]. In patients undergoing prostate radiotherapy biochemical control is improved with dose escalation [5]. However, dose escalation can also increase rectal toxicity due to the prostate - rectum proximity [6]. Although highly conformal techniques like imaged guided radiation therapy (IMRT) are able to improve dose sparing of the rectum wall, the dose to the anterior rectal wall remains high [6-8]. The dose escalation study of Kuban, et al., was able to demonstrate a direct relationship between the volume of treated rectum and rectal toxicity [6]. The Heemsbergen et al. publication on 553 evaluable patients from the Dutch dose escalation trial demonstrated a correlation between the acute toxicity and frequency of late rectal toxicity [9]. Several methods have been developed to create space between the prostate and rectum to allow for prostate dose escalation while reducing rectal wall irradiation [10-12]. One of these methods is a polyethylene glycol (PEG) hydrogel that is injected between the rectum and the prostate before treatment planning and remains stable over the treatment period. A prospective multicenter phase II study was carried out to evaluate the safety, clinical, and dosimetric effects of the hydrogel prostate-rectum spacer. Fifty-two men with localized prostate cancer were included in this trial [13,14]. In February 2013 Uhl et al. published the initial clinical outcomes with acute toxicity results of the first 48 patients and late toxicity of the first 27 patients [14]. Six patients (12%) experienced acute Grade 2 GI toxicity (no Grade 3 or 4 toxicity), while Grade 2 and Grade 3 GU toxicity was experienced in 17 (35%) and 1 (2%) of the patients, respectively. One year after the end of therapy no Grade 2 or higher GU/GI toxicity occurred. This publication presents the results from all patients following study completion, 12 months following completion of EBRT and includes results of planned proctoscopic evaluation.

## Methods

As previously described 52 men with pathologically confirmed stage T1 or T2 prostate cancer were evaluated in this prospective, non-randomized, multi-center, single arm, open-label trial. The study included otherwise healthy patients with prostates < 80 cc, PSA ≤ 20 ng/mL, Gleason Score ≤ 6 or Gleason Score 7 with a grade 3 predominant pattern. Excluded were patients with metastatic disease, planned pelvic lymph node radiotherapy, prior prostate surgery, uncontrolled diabetes, chronic systemic corticosteroid therapy, prior prostate or pelvis radiation therapy, active bleeding disorder, historical or active inflammatory bowel disease, or a history of rectal or gastrointestinal surgery. Androgen deprivation therapy was not an exclusion criteria. Following local Ethics Committee approvals patients were enrolled at the University of Heidelberg (n = 21), University of Aachen (n = 20), NKI-AVL Nederlands Kanker Instituut Amsterdam (n = 7) and University of Geneva (n = 4).

**Figure 1:**
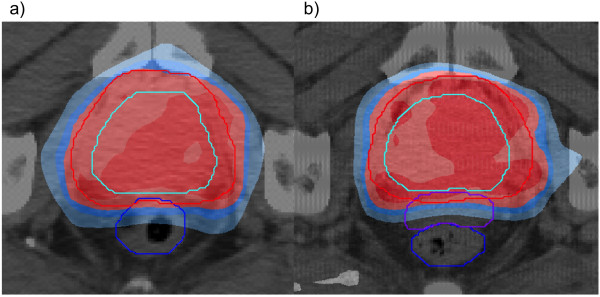
Dose distribution a) pre- and b) post injection of spacer gel.

After Informed Consent and documentation of medical/surgical history patients underwent a baseline computed tomography (CT) simulation scan to generate a baseline external beam radiation treatment plan. Subjects then underwent transperineal injection of SpaceOAR hydrogel in a procedure previously described by Hatiboglu et al. [15]. Briefly, via a transperineal approach an 18G needle was advanced using transrectal ultrasound guidance into the perirectal fat at prostate midgland, and saline was injected to expand the potential space between Denonvilliers’ Fascia and the anterior rectal wall. With the needle in the same location, 10 – 30 ml of SpaceOAR hydrogel precursors (Augmenix, Waltham, MA, USA) were injected into the same space where they polymerize within 10 seconds to form an absorbable hydrogel spacer (up to 30 ml was applied in several early patients, while the majority of patients received 10 ml). The mean procedure time for this application was 6.3 minutes [15]. After injection, a second scan for treatment planning was carried out (Figure 1). Patients then received 78 Gy of radiation delivered by IMRT technique over an 8-week period, 2 Gy per fraction, at 5 fractions per week. The clinical target volume (CTV) included the gross tumor volume (GTV) and, per the treating physician’s discretion, the proximal 2/3 of the seminal vesicles. Planning tumor volumes (PTV) included the CTV plus a 4–10 mm margin to compensate for daily setup variability and internal organ motion, with 5 mm or less posterior expansion. The guidelines for whole rectum V70 and bladder V70 tissue constraints were < 25% and < 40%, respectively. At least 99% of the PTV had to receive at least 95% of the prescription dose. A maximum dose less than 107% of the prescription dose was required. The dosimetric results of this trial were published by Song et al. [13]. Since the hydrogel produced a perirectal space ≥7.5 mm in 95.8% of the patients, the rectal V70 was reduced ≥ 25% in 95.7% of the patients, with a mean reduction of 8 Gy [13]. Acute rectal (GI) and genitourinary (GU) toxicity (RTOG/EORTC criteria as described by Cox et al.) [16] were recorded weekly during IMRT and at a visit 3 months following IMRT. Late GI and GU toxicity was similarly assessed at visits 6 and 12 months following IMRT completion. Additionally at 6 months post IMRT patients underwent MRI scans to assess hydrogel absorption, and at 12 months post IMRT, PSA levels were measured and proctoscopy was performed. Proctoscopic observations of congested mucosa, telangiectasia, ulceration, stricture and necrosis were scored using the Vienna Rectoscopy Scale [17].

## Results

The average age of patients enrolled in this study was 68.9 ± 8.0 years, with the average time since initial prostate cancer diagnosis being 110 ± 140 days. Fifty four percent of the patients had T1 stage (2% T_1a_, 2% T_1b_, 50% T_1c_), with the remaining having T2 stage (6% T_2,_ 17% T_2a_, 17% T_2b_, 6% T_2c_). Average patient PSA was 6.9 (range 0.1 - 19.8), with Gleason score 6 and 7 (Grade 3 predominant pattern) being 52% and 48%, respectively. Prostate volume in study patients was 56.9 ± 20.4 cc. There were four patients excluded from the per-protocol population (n = 48) due to no hydrogel injection (n = 2), inadvertent rectal wall injection (n = 1) and improper polymer reconstitution (n = 1). These events took place early in the study and were addressed with previously published procedural modifications [14]. The rectal wall injection resulted in focal rectal mucosal necrosis which completely resolved with no further sequelae, while the improper polymer reconstitution resulted in no gel formation and no patient complications. Additionally, one patient withdrew from the study resulting in 47 patients in the late follow up population. These 12 months post treatment follow up toxicity results represent the final evaluation of previously published collective. A total of 19 (39.6%) and 6 (12.5%) patients experienced acute Grade 1 and Grade 2 GI toxicity, respectively (Table 1). There was no Grade 3 or Grade 4 acute GI toxicity experienced in the study. A total of 45 (95.7%) patients experienced no late GI toxicity (95.7%), with 2 (4.3%) patients experiencing late Grade 1 GI toxicity. There was no late Grade 2 or greater GI toxicity experienced in the study. A total of 20 (41.7%), 17 (35.4%) and 1 (2.1%) patients experienced acute Grade 1, Grade 2 and Grade 3 GU toxicity, respectively (Table 1). There was no Grade 4 acute GU toxicity experienced in the study. A total of 8 (17.0%) and 1 (2.1%) patients experienced late Grade 1 and Grade 2 GU toxicity, respectively. There was no late Grade 3 or greater GU toxicity experienced in the study. The mean prostate to rectum distance at prostate midgland was 9.7 ± 5.5 mm following hydrogel injection, 10.5 ± 5.3 mm following completion of IMRT, and 2.9 ± 4.2 mm three months following completion of IMRT, reflecting hydrogel absorption. MRI scans to assess hydrogel absorption were obtained in 44 patients 6 months following IMRT. Aside from a small amount of absorbing gel in one patient (2.3%), hydrogel was found to be completely absorbed in every case. Forty-five (45) of 47 patients (95.7%) in the Per-Protocol Population underwent proctoscopic exams at 12 months following completion of EBRT. Of the 45 subjects evaluated, 32 patients (71%) had a VRS score of 0. Grade 2 congested mucosa was noted for 1 subject (3%) and telangiectasia was found in 28% of subjects: 13% Grade 1, 13% Grade 2, and 2% for Grade 3. There was no evidence of ulceration, stricture or necrosis at 12 months. PSA values at 12 months post EBRT are available for 45 subjects. Every patient showed a decreasing PSA value after treatment. No incidence of PSA relapse could be observed one year after irradiation. The mean PSA at 12 months post EBRT was 0.99 ± 0.09 ng/mL, which represents a decrease of 5.87 ± 4.23 ng/mL compared to baseline.

**Table 1:** Acute and late GI/GU toxicity (Maximum score)

**Grade**	**GI toxicity scores (n %)**		**GU toxicity scores (n %)**	
	**Acute**	**Late**	**Acute**	**Late**
0	23 (48.0%)	45 (95.7%)	10 (21.0%)	38 (80.9%)
1	19 (39.6%)^1^	2 (4.3%)^2^	20 (41.7%)^3^	8 (17.0%)^4^
2	6 (12.5%)	0 (0%)	17 (35.4%)^5^	1 (2.1%)
3	0 (0%)	0 (0%)	1 (2.1%)	0 (0%)
4	0 (0%)	0 (0%)	0 (0%)	0 (0%)
Grade 1 or worse	25 (52.1%)	2 (4.3%)	38 (79.2%)	9 (19.1%)
Grade 2 or worse	6 (12.5%)	0 (0%)	18 (37.5%)	1 (2.1%)

^1^1 subject was Grade 1 at Baseline, ^2^1 subject was Grade 1 at Baseline, ^3^3 subjects were Grade 1 at Baseline, ^4^6 subjects were Grade 1 at Baseline, ^5^3 subjects were Grade 1 at Baseline, 1 subject was Grade 2 at Baseline.

## Discussion

**Figure 2:**
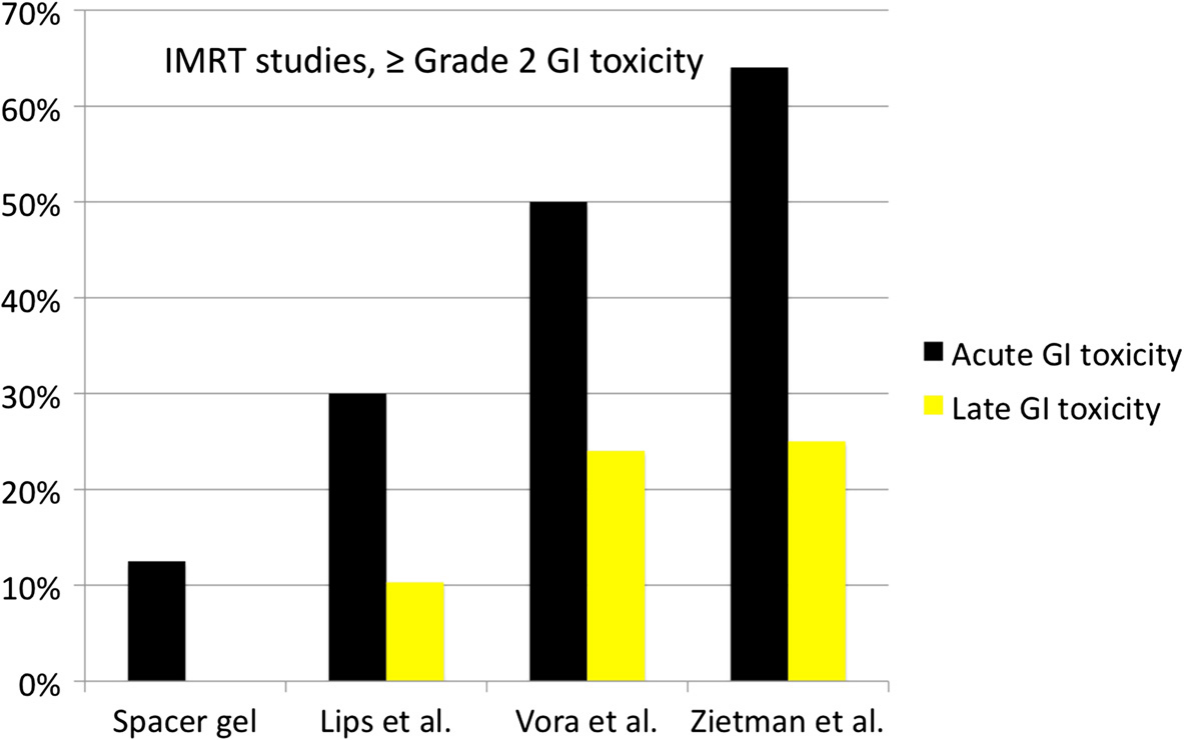
**Comparison of gastrointestinal toxicity ≥ Grade 2 with other trials.** Lips et al. [18]: PTV = Prostate + seminal vesicles + 8 mm margin, 76 Gy mean dose, not more than 5% of rectum received ≥ 72 Gy. Vora et al. [19]: PTV = Prostate + seminal vesicles + 6-10 mm margin, 50.4 Gy + Boost (median 75.6 Gy), not more than 40%/30%/10% of Rectum received ≥ 65 Gy/70 Gy/75 Gy, not more than 1.8 cm^2^ of rectum received 81 Gy. Zietman et al. [8]: PTV = Prostate + seminal vesicles + 10 mm margin for Photontherapy (50.4 Gy) and 5mm margin for proton Boost (28.8 GyE).

**Figure 3:**
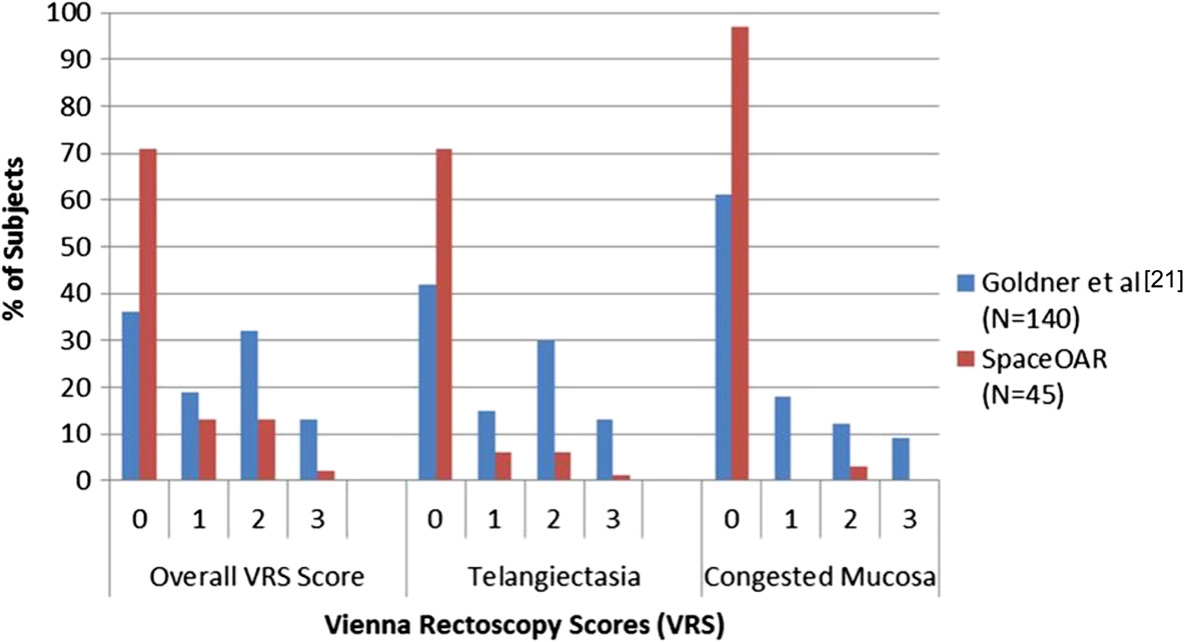
**Comparison of Vienna rectoscopy scores at 12 months for men treated with SpaceOAR vs. the literature [**[21]**].**

While studies have demonstrated that dose escalation improves local control in men with prostate cancer, concerns of rectal toxicity limits implementation [6,8]. Thus, a meaningful dose escalation is only possible with a better sparing of rectal tissue. Conformal techniques such as brachytherapy, IMRT and proton therapy are helping to resolve this problem. Despite improvements in dose conformity, intra-fraction prostate motion can move the anterior rectum into the high dose region. A reduction of the irradiated volume posterior to the prostate is not a good solution, since most prostate cancers in the peripheral zone of the gland, occur adjacent to the rectum. A very simple and logical solution is to create more distance between the required volume to be irradiated and the anterior portion of the prostate. This can be easily achieved with the injection of a spacer between the rectum and prostate to create and maintain space throughout treatment. The feasibility and effectiveness of the hydrogel injection were objectives of this study. It has already been demonstrated that the injection procedure is safe and a stable 1 cm distance between the prostate and the rectum can be generated [15] resulting in a significant dose reduction to the rectum [13]. The prospectively collected data show a very low GI acute toxicity with only 12.5% Grade 2, and no Grade 3 or higher toxicity. A total of 95.7% of patients had no late GI toxicity and only 4.3% (n = 2) had late Grade 1 GI toxicity. After completion of the follow up time, these results validate our published data with early results [14]. Despite some differences in margins and dose delivered, the lower GI toxicity rates in this study are remarkable when compared to other studies (Figure 2) [8,18,19]. Like a number of other toxicity reports, proctoscopy was also performed 12 months after the end of therapy [17]. Ippolito et al. could show that early proctoscopy 12 months after irradiation can be used as a surrogate endpoint for late rectal toxicity. The incidence of late rectal toxicity ≥ grade 2 was higher in patients with VRS score grade ≥2 or 3 [20]. In our results no evidence of ulceration, stricture or necrosis was found. Seventy one percent of patients had a VRS score 0, with 13% and 2% having Grade 2 and Grade 3, respectively. Another prospective multicenter trial demonstrated a direct correlation between VRS and EORTC/RTOG score 12 months after prostate irradiation with 70 or 74 Gy [21]. At 12 months following IMRT the pathological changes to rectum mucosa in this hydrogel spacer trial are less that in the Goldner et al. prospective trial, despite the higher radiation dose in this hydrogel spacer study (Figure 3). Other studies with spacer between the rectum and prostate show similar toxicity reductions. Noyes et al. evaluated human collagen injections into the perirectal space and found a subsequent reduction of GI toxicity in patients compared with a historical control group [10]. Wilder published similar results after hyaluronic acid injection [12]. The PEG gel in our study was stable during treatment and was reabsorbed within a year. The patients in this hydrogel trial experienced 41.7%/35.4%/2.1% Grade 1/2/3 acute GU toxicity, respectively. A total of 35% and 2% had Grade 1 and Grade 2 GU toxicity at 12 months after treatment. No ≥ Grade 3 GU toxicity was experienced in this trial. Thus, the rate of patients with GU toxicity ≥ 2 is favorable compared to studies without spacers [8,18,19]. Despite the added cost of the product, routine incorporation of hydrogel may result in significant overall health system savings as a result of decreased toxicity and less frequent need for proctitis treatment, fewer treatment fractions (hypofractionation) may be made even more safe and, potentially, a lower rate of cancer recurrence (dose escalation).

## Conclusion

The use of PEG spacer gel is a safe and effective method to spare the rectum from higher dose and toxicity. Due to fewer late side effects on the rectum, along with the potential of enabling hypofractionation and dose escalation, the hydrogel spacer may lead to lower costs for the healthcare system.

## Competing interests

Financial and material support

This study was supported by Augmenix, Inc.

Research funding

This study was partially (Dr. K. Herfarth) funded by German Research foundation DFG (KFO214; He2499/3-1).

Financial disclosures

Dr. DeWeese served as medical monitor for this study while Dr. Song served as Independent Data Reviewer. Both received compensation for these activities.

Honoraria

BrainLab (Dr. Weber).

## Authors’ contributions

MU, KH, ME, MP, BT, RK, DW, RM, DS and TD participated in the design of the study and reviewed the results. MU, KH, ME, MP, BT, RK, DW and RM were responsible for the patient recruitment. MU, KH, ME, MP, BT, RK, DW and RM performed planning and radiation therapy. DS and TD were responsible for the statistical analysis. MU drafted the manuscript. All authors read and approved the final manuscript.
